# Editorial: Recent Advances in Potential Biomarkers for Rheumatic Diseases and in Cell-Based Therapies in the Management of Inflammatory Rheumatic Diseases

**DOI:** 10.3389/fimmu.2021.836119

**Published:** 2022-01-12

**Authors:** Philippe Saas, Eric Toussirot, Katarzyna Bogunia-Kubik

**Affiliations:** ^1^ Univ. Bourgogne Franche-Comté, INSERM, EFS BFC, UMR1098 RIGHT, Interactions Hôte-Greffon-Tumeur/Ingénierie Cellulaire et Génique, Fédération Hospitalo-Universitaire INCREASE, LabEx LipSTIC, Besançon, France; ^2^ INSERM CIC-1431, Centre d’Investigation Clinique Biothérapie, Pôle Recherche, CHU de Besançon, Besançon, France; ^3^ Fédération Hospitalo-Universitaire INCREASE, CHU de Besançon, Besançon, France; ^4^ Rhumatologie, Pôle PACTE (Pathologies Aiguës Chroniques Transplantation Éducation), CHU de Besançon, Besançon, France; ^5^ Département Universitaire de Thérapeutique, Université de Bourgogne Franche-Comté, Besançon, France; ^6^ Hirszfeld Institute of Immunology and Experimental Therapy, Polish Academy of Sciences, Wroclaw, Poland

**Keywords:** inflammatory rheumatic diseases, spondyloarthritis, rheumatoid arthritis, systemic lupus erythematosus, psoriatic arthritis, biomarkers, TNF inhibitors, cell-based therapy

Inflammatory rheumatic diseases (IRD) constitute a wide spectrum of disorders encompassing inflammatory arthropathies, such as rheumatoid arthritis (RA), axial spondyloarthritis (ax-SpA) and psoriatic arthritis (PsA). It also includes connective tissue disorders, like systemic lupus erythematosus (SLE), Sjögren’s syndrome (SS) or systemic sclerosis (SSc). This highly heterogeneous group of conditions has multifactorial and not fully understood etiology. It is characterized by the presence of persistent inflammation affecting primarily the musculoskeletal system and connective tissue. Disease progression ultimately leads to organ damage, functional disability, premature mortality, as well as economic and social burdens. Rheumatoid arthritis and SpA represent the most common IRD. The management of RA, and other forms of IRD, has dramatically changed in the last 20 years with the development of targeted therapeutic agents, namely biological disease-modifying anti-rheumatic drugs (bDMARD) and targeted synthetic disease-modifying anti-rheumatic drugs (tsDMARD). However, a substantial proportion of patients still exhibits an inadequate response, does not achieve remission, or develop undesirable side effects (1). This is well illustrated for TNF inhibitors (TNFi) in RA or SpA (2) with a well-known risk of infections.

Biomarkers constitute a powerful approach to the diagnosis and management of IRD. Rheumatoid factor (RF) and anti-citrullinated peptide antibodies (ACPA) are the most well-established markers for RA diagnosis and prognosis. The presence of antinuclear antibodies (ANA) – that are directed against intracellular antigens – is the hallmark of SLE and is found in other connective tissue disorders. Moreover, one of the most significant genetic markers for IRD is human leukocyte antigen (HLA) HLA-B27 allele, which is strongly associated with ax-SpA. In recent years, a novel multi-biomarker disease activity (MBDA) test for disease activity assessment in RA patients has been developed and implemented into clinical practice. This blood-based test measures the levels of 12 serum proteins and a composite MBDA score is calculated from a combination of these measures. This MBDA score correlates with Disease Activity Score (DAS), DAS28 and C reactive protein (CRP) (3). It is also a predictor for radiographic progression (4).

Rapid progress in molecular biology during the last two decades has resulted in developments in the field of biomarker research. These advances can lead to the improvement of disease prevention and early detection, as well as significant progress in the assessment of disease activity or prognosis for individual patients. Furthermore, biomarkers can be used as powerful tools to predict the response to therapy, establish the optimal therapeutic dose, and monitor the treatment efficacy. This progress can also be applied to develop new therapeutic approaches, such as cell-based therapies.

Cell therapy is one therapeutic approach of IRD, based on the administration of cells that can control the immune deregulation, inflammatory cytokine production and overall systemic inflammation. This can ultimately prevent joint damage. A large body of clinical researches has helped to describe the potential immune-modulating properties and regenerative potential of cell-based therapies.

The initial objective of this current Research Topic was to collect original research and review manuscripts dealing with biomarkers and cell-based therapies in IRD. Among the 27 manuscripts composing this Research Topic, 20 deal with biomarkers and three with cell-based therapies. As handling editors, we decided to accept one manuscript reporting a new animal model for RA, another one consisting in the description of a fully automated method for routine diagnosis, and two manuscripts on new therapeutic strategies. Indeed, RA animal models have been shown to be precious for the development of new therapies that are now used routinely in RA, such as TNFi (5). The advantage of the new animal model proposed by Zhao et al. is to use C57BL/6 mice for which numerous genetically modified strains exist. For this purpose, the authors immunized C57BL/6NJ mice with human cartilage oligomeric matrix protein. This induced a severe arthritis with a high incidence, associated with strong autoantibody (auto-Ab) responses. Thus, this model will be useful to test new therapeutic strategies for RA patients. Trained immunity, defined as immunological memory of innate cells including dendritic cells (DC), macrophages or NK cells, is associated with changes in cell metabolism and epigenetic reprogramming. Municio and Criado discuss drugs that target glycolysis, lipid metabolism or epigenetic reprogramming to treat IRD, like RA or SLE. Among these drugs, we want to highlight new biologics targeting GM-CSF or its receptor - a critical signaling pathway responsible for trained immunity - that are currently tested in clinical trials enrolling RA or PsA patients. Yan Q. et al. reported that Tofacitinib - an oral, small-molecule Janus kinase (JAK) inhibitor used in RA and ulcerative colitis - can be a potential new tsDMARD in SLE, since it ameliorates experimental SLE by suppressing T cell activation through interactions with TGF-beta type I receptor. Besides these new potential treatments, three reviews discuss cell-based therapies mainly in RA. Two deal with mesenchymal stroma/stem cells (MSC). Hwang et al. summarized nicely recent advances in clinical studies using MSC for the treatment of RA and the degenerative joint disease, osteoarthritis (OA). These authors recalled that MSC have led to a significant number of clinical trials in a wide range of diseases; it is estimated around more than thousand trials involving around fifty thousand patients between 2011 and 2018. Ten MSC treatments are approved by regulatory authorities in different indications in the world. Among these treatments, one using human umbilical cord blood (UCB)-derived MSC has been approved in South Korea for knee articular cartilage defects in OA. These authors discussed the seven phase I/II trials performed in the world on OA and the three on RA. They evoked also the four different sources of MSC and their specific features, namely bone marrow, adipose tissue, UCB and synovium. The second manuscript on MSC by El-Jawhari et al. is not only focused on the results of 16 phase I/II clinical trials performed in RA. The authors discussed also the functions of MSC in healthy joints and how these functions are altered in RA and SLE. They mentioned also the interactions of MSC with immune cells involved in these two conditions. In addition, they evoked the future for MSC treatment, *i.e*., non-cellular extracellular vesicles derived from MSC (a current intense field of research) and apoptotic MSC. This makes a link with the last review on cell-based therapies. Before presenting this review, we want to highlight two recent works showing that the therapeutic effects of MSC may be related to their apoptosis in the lungs after intravenous administration and subsequent efferocytosis (*i.e*., the non-inflammatory removal of apoptotic cells by professional phagocytes) (6, 7). Toussirot et al. summarized experimental studies suggesting the potential therapeutic properties of apoptotic leukocytes in RA. This approach involves also *in vivo* efferocytosis. Whether a common mechanism exists between these two cell-based therapies is still provocative and remains to be determined.

Concerning the manuscripts dealing with biomarkers received in the setting of this Research Topic, we propose to consider them depending on their aims; that is, those evaluating disease activity or severity (n=16), those predicting disease complications (n=1) and those appreciating the response to treatment (here, exclusively TNFi, n=3). As mentioned before one of the manuscripts is on routine diagnosis: Choi et al. evaluated an analyzer called Helios to fully automate ANA indirect immunofluorescence assay in a real-life laboratory setting. While this analyzer allows labor, time, and cost savings, it requires additional validation of the results by biologists. Biomarkers used to evaluate disease activity or severity can be classified into three groups: (i) plasma proteins including auto-antigens (auto-Ag) and Auto-Ab, (ii) biomarkers assessed by molecular biology including single nucleotide polymorphisms (SNP) and circular RNA (circRNA), and finally (iii) approaches to analyze pathogenic cells. These three biomarker groups will be evoked below in different paragraphs.

Among the six manuscripts concerning proteins as biomarkers of disease activity or severity, the first one by Hosman et al. is an extensive review on a well-known inflammatory marker, serum amyloid A (SAA), in several IRD. Several tables summarize the clinical utility of SAA in RA, ax-SpA, PsA, SLE or SSc. We can then gather three manuscripts dealing with RA and one of its associated neo-auto-Ag generated by the post-translational modification of protein arginine residues by PeptidylArginine Deiminases (PAD), namely citrullinated proteins. Won et al. reported that a monoclonal antibody (mAb) specific to citrullinated peptide, called 12G1, is able to detect citrullinated type II collagen, and filaggrin. This mAb may be useful for diagnosis of RA including RA patients who are negative for both RF and ACPA. Bezuidenhout et al. and Larid et al. focused on citrullinated fibrin. The first work illustrated the tight relationship between activation of the immune system and the procoagulatory status of patients with IRD, and in particular RA (8). Indeed, Bezuidenhout et al. showed the citrullination of fibrin in plasma clots of RA patients and its relationship with prothrombotic tendency in these patients. Thus, citrullinated fibrin(ogen) may play a role in the thrombotic risk of RA patients. Larid et al. identify a citrullinated fibrin(ogen) peptide (α501-515cit) and high levels of circulating auto-Ab directed against this α501–515cit epitope that could be involved in the development of rheumatoid nodules in RA patients expressing the HLA-DRB1*04:01 allele. Now moving from RA to other IRD, Martin et al. reported that circulating C4d correlates with C4d deposition in the kidneys of lupus nephritis patients and the usefulness of this circulating biomarker to evaluate treatment response in this disease. After targeted analysis of auto-Ag, auto-Ab and complement system proteins, Padern et al. used a non-targeted approach, namely proteomic to identify potential biomarkers in primary SS patients compared to RA and SLE patients. Two biomarker combinations allowed the authors to discriminate primary SS patients from RA and SLE patients, namely the low concentrations of BDNF and Fractalkine/CX3CL1 and high concentrations of I-TAC/CXCL11 associated with low concentrations of sCD163. Whether all these biomarkers will be useful in large patient cohorts and in routine patient follow-up remains to be determined.

We mentioned previously the huge progress made in molecular biology and its contribution in the fields of biomarkers. Two original papers identified SNP in IRD. Braga et al. reported that *IL10* (rs1800896) polymorphism influences TNF-α, IL-10, IL-17A and IL-17F serum levels in Caucasian patients with ax-SpA. Huang et al. found the association of different *CD40* gene polymorphisms (rs3765456, rs1569723, rs73115010, rs13040307, rs1883832 and rs4810485) with SLE and RA in a Chinese Han population. A review by Liu et al. introduced circRNA. This corresponds to a novel class of endogenous non-coding RNA that regulate gene expression and transcription. Expression patterns of circRNA are tissue- and disease-specific. Cai et al. report that a particular circRNA, named Circ_0088194, promotes the invasion and migration of RA fibroblast-like synoviocytes *via* the miR-766-3p/MMP2 axis. The last manuscript by Hong et al. used single cell RNA sequencing and a confirmation by flow cytometry to identify two CD4^+^ T cell subsets that expand in primary SS patients. This consists in cytotoxic granzyme B^+^ CD4^+^ T cells and CD4^+^ T cells expressing a specific TCR alpha chain, TRAV13-2^+^. This may constitute potential new therapeutic targets of primary SS.

Analysis of pathogenic cells or the factors released by these cells may also allow the identification of new biomarkers or of potential new treatment targets. Among the five manuscripts analyzing pathogenic cells or factors, one concerns adaptive immune cells and more specifically CD4^+^ T cell subsets, while the other four studies consider both resident and circulating innate immune cells *(i.e*., synovial fibroblasts and macrophages *versus* myeloid-derived suppressor cells [MDSC]). Paradowska-Gorycka et al. analyzed transcription factors, Th17/FoxP3^+^ CD4^+^ regulatory T cells (Treg) and circulating cytokines in RA and OA patients. They identified SMAD3 and STAT3 as potential diagnostic biomarkers of RA. Nakano et al. studied the role of canonical NF-κB signaling in synovial fibroblasts, and more particularly in IL-1β-induced COX-2 expression. The work of Fuentelsaz-Romero et al. focused on macrophages and synovial fibroblasts present in the synovial tissues of patients with RA, PsA and undifferentiated arthritis (UA). They concluded that GM-CSF is highly expressed by sublining CD90^+^ FAP^+^ synovial fibroblasts, and CD163^+^ macrophages and that this cytokine may be a potential therapeutic target in RA, PsA and UA. This makes a link with the review of Municio and Criado. that we previously quoted. Yan L et al. summarized in a review the pro- and anti-inflammatory properties of MDSC - initially identified in cancer - in the setting of RA and in experimental RA models. They evoked the two main subsets, monocytic MDSC and granulocytic MDSC (also termed polymorphonuclear [PMN]-MDSC). Zhong et al. illustrated this ambivalent role of MDSC in the inflammatory joint disease, the gout. A higher frequency of circulating PMN-MDC was found in patients with gout in comparison with healthy controls (HC). These circulating PMN-MDC levels correlated with pathological indicators, namely uric acid and CRP. These cells exert stronger immune suppressive functions illustrating the anti-inflammatory properties of MDSC, but they produced high levels of IL-1β in response to monosodium urate. Thus, MDSC may be a potential new target for gout treatment. Secondary hemophagocytic lymphohistiocytosis/macrophage activation syndrome (sHLH/MAS) may occur as a complication of IRD (9). Pascarella et al. studied monocytes from sHLH/MAS. They observed higher basal levels of phosphorylated STAT1, as well as a higher response of these cells to IFN-γ stimulation. However, since glucocorticoids - one of the standard treatment of sHLH/MAS - affect the phosphorylation STAT1 levels, phosphorylated STAT1 levels could not be considered as a useful biomarker.

In this paragraph, we will summarize the works on biomarkers predicting the response to TNFi mainly in RA. Despite significant improvements in RA treatment brought by bDMARD and in particular TNFi, a substantial proportion of patients does not respond adequately to these drugs and, some of them do not achieve remission or low disease activity (10, 11), which is the therapeutic goals in RA. Thus, the identification of biomarkers that may predict the response to bDMARD including TNFi is of high interest. Three manuscripts in this Research Topic proposed to identify predictive biomarkers for response to TNFi with three different approaches. In a study analyzing *IL-33* gene SNP in a Caucasian population, Iwaszko et al. identified *IL-33* rs16924159 SNP as associated with TNFi efficacy and clinical parameters in RA and ax-SpA patients and rs10975519 with RA risk in females. The authors conclude that additional studies with larger sample sizes and different populations are needed to validate these findings. The approaches used by the other two studies are more complex and analyze a huge number of clinical and/or biological parameters. Sánchez-Maldonado et al. performed a genome-wide association study (GWAS) in two large cohorts of RA patients, a discovery and a verification cohort (including 1361 and 706 RA patients, respectively). These authors confirmed the association of the *LINC02549_rs7767069_* SNP with a poor response to TNFi, appreciated by the DAS28. *LINC02549* (Long Intergenic Protein Coding RNA 2549) is an RNA gene affiliated with the long non-codding RNA. A RF-stratified analysis also showed a RF-specific association for the *LRRC55_rs717117_* SNP (encoding the leucine-rich repeat-containing protein 55) with response to TNFi. Luque-Tévar et al. performed the integrative analysis of clinical and molecular parameters using machine-learning algorithms. This prospective multicenter and longitudinal study enrolled 104 patients from two independent cohorts and 29 HC. Serum inflammatory parameters, oxidative stress markers, and NETosis-derived bioproducts as well as regulating microRNA were analyzed. Based on these parameters, the authors identified three clusters: cluster 1 with patients exhibiting a medium DAS and low radiologic involvement, cluster 3 comprised patients with highest DAS, and cluster 2 representing patients with an intermediate clinical phenotype. Six months after TNFi therapy, all patients in cluster 1 demonstrated a clinical response and few or no changes in the analyzed parameters. However, these patients exhibited a less prominent inflammatory status at baseline. Non-responders to TNFi were found in cluster 2 and 3. Patients from these clusters responding to TNFi exhibited a significant reduction in levels of inflammatory parameters, oxidative stress markers, and products of NETosis associated with the restoration in the levels of microRNA. On the opposite, TNFi resistant patients did not demonstrate these changes. Validation in routine follow-up is required to appreciate the usefulness of these biomarkers.

Overall, this Research Topic has been very stimulating for us as handling editors and we hope that it will be useful for researchers and physicians working in the fields of IRD, and in particular biomarker identification and development of cell-based therapies. We would like to finish by thanking the authors and the reviewers.

## Author Contributions

All authors listed have made a substantial, direct and intellectual contribution to the work and approved it for publication.

## Funding

PS is supported by the Agence Nationale de la Recherche (ANR-11-LABX-0021 to Labex LipSTIC), the the Conseil Régional de Franche-Comté (“Soutien au LabEX LipSTIC”), and by the MiMedI project funded by BPI France (grant No. DOS0060162/00), and the European Union through the European Regional Development Fund of the Region Bourgogne-Franche-Comte (grant No. FC0013440). ET is supported by Programme Hospitalier de Recherche clinique PHRC-I|_ GIRCI_ EST_B02 2015. KB-K was supported by the National Science Centre, Poland (grant No. 2016/21/B/NZ5/01901).

## Conflict of Interest

PS is the author of a patent and a shareholder of Med’Inn’Pharma, related to the development of anti-inflammatory treatment.

The remaining authors declare that the research was conducted in the absence of any commercial or financial relationships that could be construed as a potential conflict of interest.

## Publisher’s Note

All claims expressed in this article are solely those of the authors and do not necessarily represent those of their affiliated organizations, or those of the publisher, the editors and the reviewers. Any product that may be evaluated in this article, or claim that may be made by its manufacturer, is not guaranteed or endorsed by the publisher.
